# Synthesis of Ultrahigh Molecular Weight Polymers with Low PDIs by Polymerizations of 1-Decene, 1-Dodecene, and 1-Tetradecene by Cp*TiMe_2_(O-2,6-*^i^*Pr_2_C_6_H_3_)–Borate Catalyst

**DOI:** 10.3390/molecules24081634

**Published:** 2019-04-25

**Authors:** Kotohiro Nomura, Sarntamon Pengoubol, Wannida Apisuk

**Affiliations:** Department of Chemistry, Tokyo Metropolitan University, 1-1 Minami Osawa, Hachioji, Tokyo 192-0397, Japan; sarntamon@gmail.com (S.P.); wannida.ppc@gmail.com (W.A.)

**Keywords:** polymerization, titanium complex, catalyst, α-olefin, half-titanocene, borate

## Abstract

Polymerizations of 1-decene (DC), 1-dodecene (DD), and 1-tetradecene (TD) by Cp*TiMe_2_(O-2,6-*^i^*Pr_2_C_6_H_3_) (**1**)–[Ph_3_C][B(C_6_F_5_)_4_] (borate) catalyst have been explored in the presence of Al cocatalyst. The polymerizations of DC and DD, in *n*-hexane containing a mixture of Al*^i^*Bu_3_ and Al(*n*-C_8_H_17_)_3_, proceeded with high catalytic activities in a quasi-living manner, affording high molecular weight polymers (activity 4120–5860 kg-poly(DC)/mol-Ti·h, *M*_n_ for poly(DC) = 7.04–7.82 × 10^5^, after 20 min at −30 °C). The PDI (*M*_w_/*M*_n_) values in the resultant polymers decreased upon increasing the ratio of Al(*n*-C_8_H_17_)_3_/Al*^i^*Bu_3_ with decreasing the activities at −30 °C. The PDI values also became low when these polymerizations were conducted at low temperatures (−40 or −50 °C); high molecular weight poly(DD) with low PDI (*M*_n_ = 5.26 × 10^5^, *M*_w_/*M*_n_ = 1.16) was obtained at −50 °C. The TD polymerization using **1**–borate–Al*^i^*Bu_3_ catalyst (conducted in *n*-hexane at −30 °C) afforded ultrahigh molecular weight poly(TD) (*M*_n_ = 1.02 × 10^6^, *M*_w_/*M*_n_ = 1.38), and the PDI values also decreased with increasing the Al(*n*-C_8_H_17_)_3_/Al*^i^*Bu_3_ ratio.

## 1. Introduction

Design of molecular catalysts for olefin polymerization has been considered as an important subject in synthesis of new polymers with specified functions. The recent progress in the catalyst developments provides new possibilities [1,2,3,4,5,6,7,8,9,10,11,12,13,14,15,16,17]. Although crystalline isotactic polypropylene has been widely used in our daily life, use of amorphous poly(α-olefin)s (APAOs) showed less attention due to their inherent stickiness and softness. APAOs possess high melt-flow rate with low density, and are used in hot melt applications, these also improve adhesion on wood and polypropylene, and improve the free-flowing ability of their granules. It has been known recently that ultrahigh molecular weight poly(α-olefin)s can be used as drag reducing agents (DRAs) in pipeline transport methods for crude oil and petroleum products, because of their ability to reduce pumping power and increase piping system capacity [18,19,20,21]. Moreover, poly(α-olefin)s with alkyl chain length greater than six have bottlebrush architecture (branched macromolecules with a high graft density along their backbone) [22,23,24,25], and are the simplest bottlebrush polymers, with their backbone and side chains consisting of alkanes.

As shown below (Table 1), polymerization of α-olefin (1-hexene, 1-octene, 1-decene, etc.) by ordinary metallocene catalysts gave oligomers [26,27,28]. Several examples [28,29,30,31,32,33] were known for synthesis of (ultra)high molecular weight poly(1-hexene)s by using [2,2-(O-4-Me-6-*^t^*Bu-C_6_H_3_)_2_S]TiCl_2_ (in the presence of specified modified MMAO) [31,32], or (C_5_HMe_4_)_2_HfCl_2_ (under ultrahigh pressure) [29]. One example was known for synthesis of isotactic poly(1-hexene)s with ultrahigh molecular weights by titanium complexes containing diamine bis(phenolate) ligands [30]. Several examples were also known for synthesis of poly(α-olefin)s with rather high molecular weights [27,34,35,36,37,38,39,40,41,42]. However, synthesis of ultrahigh molecular weight polymers by polymerization of higher α-olefins (1-decene, 1-dodecene, 1-tetradecene, etc.) still have been limited [28], probably due to their tendency to undergo β-hydrogen elimination before subsequent/repeated insertion.

**Scheme 1:**
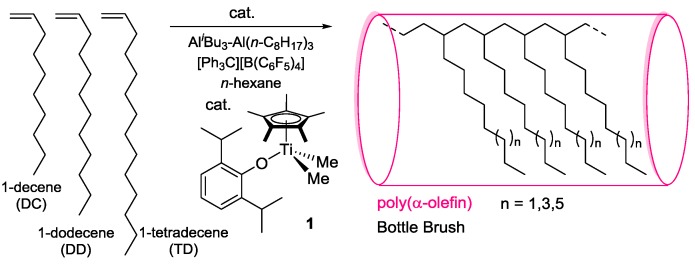
Polymerization of 1-decene (DC), 1-dodecene (DD), and 1-tetradecene (TD) using Cp*TiMe_2_(O-2,6-*^i^*Pr_2_C_6_H_3_) (**1**)–[Ph_3_C][B(C_6_F_5_)_4_] catalyst in the presence of Al cocatalyst.

We recently reported synthesis of high molecular weight poly(α-olefin)s by polymerizations of 1-decene, 1-dodecene, 1-hexadecene, and 1-octadecene by Cp*TiCl_2_(O-2,6-*^i^*Pr_2_C_6_H_3_)–MAO catalyst [28], and the *M*_n_ values in the resultant polymers were higher than those prepared by [Me_2_Si(C_5_Me_4_)(N*^t^*Bu)]TiCl_2_ and Cp_2_ZrCl_2_ (Table 1) [28]. Moreover, Cp*TiMe_2_(O-2,6-*^i^*Pr_2_C_6_H_3_) (**1**)–[Ph_3_C][B(C_6_F_5_)_4_] (borate) catalyst showed the higher catalytic activities, affording ultrahigh molecular weight poly(1-octene)s and poly(1-dodecene)s (Table 1) [28]. On the other hand, we also reported that 1-hexene polymerization by **1**–borate catalyst proceeded in a quasi-living manner under certain conditions to afford ultrahigh molecular weight poly(1-hexene)s (*M*_n_ ≥ 1.0–1.9 × 10^6^) [33]. Therefore, we herein present synthesis of high molecular weight polymers by polymerizations of 1-decene (DC), 1-dodecene (DD), and 1-tetradecene (TD) with low PDI (*M*_w_/*M*_n_) values using **1**-borate catalyst in the presence of Al cocatalyst (Scheme 1). 

## 2. Results and Discussion

### 2.1. Polymerization of 1-Decene and 1-Dodecene by Cp*TiMe_2_(O-2,6-^i^Pr_2_C_6_H_3_) (1)–Borate Catalyst

On the basis of our previous report in 1-hexene polymerization [33], polymerizations of 1-decene (DC) using Cp*TiMe_2_(O-2,6-^i^Pr_2_C_6_H_3_) (**1**)–[Ph_3_C][B(C_6_F_5_)_4_] (borate) catalyst were conducted in *n*-hexane in the presence of Al cocatalyst (Al/Ti = 500, molar ratio) with different Al(*n*-C_8_H_17_)_3_/Al*^i^*Bu_3_ ratios at −30 °C. Use of Al(*n*-C_8_H_17_)_3_, which should be the weak reagent for alkylation and/or chain transfer, but plays a role as a scavenger, was effective in the 1-hexene polymerization to proceed without catalyst deactivation [33]. The similar effect was also observed in the syndiospecific styrene polymerization using (*^t^*BuC_5_H_4_)TiCl_2_(O-2,6-*^i^*Pr_2_C_6_H_3_)–[PhN(H)Me_2_]-[B(C_6_F_5_)_4_] catalyst [43]. Use of a mixture of Al(*n*-C_8_H_17_)_3_/Al*^i^*Bu_3_ cocatalyst was also effective for exclusive obtainment of copolymers with high styrene contents in the ethylene/styrene copolymerization even at high temperature [44,45]. It was assumed that the Al alkyl would also play a role to stabilize the catalytically active species for the subsequent decomposition by reacting with borate [46,47,48]. 

**Figure 1:**
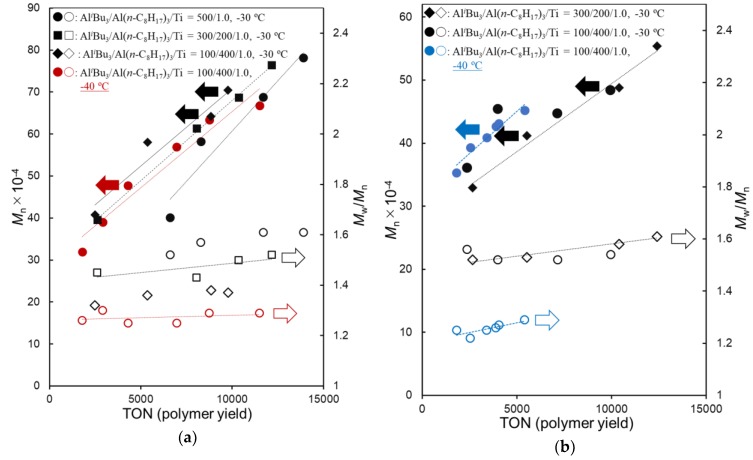
Plots of *M*_n_, *M*_w_/*M*_n_ vs. polymer yields (turnover numbers, TON) in (**a**) 1-decene (DC) and (**b**) 1-dodecene (DD) polymerization using Cp*TiMe_2_(O-2,6-*^i^*Pr_2_C_6_H_3_) (**1**)–borate catalyst. Detailed data are shown in Table 2 and Table 3.

As shown in Table 2, the polymerization of DC at −30 °C (runs 1–3) proceeded with high catalytic activities (4120–5860 kg-polymer/mol-Ti·h after 20 min) without significant catalyst deactivation to afford high molecular weight poly(DC)s (*M*_n_ = 7.04–7.82 × 10^5^), and the *M*_n_ values increased over time course without increasing the PDI (*M*_w_/*M*_n_) values. It turned out that the PDI values decreased upon increasing the Al(*n*-C_8_H_17_)_3_/Al*^i^*Bu_3_ molar ratio, whereas the catalytic activity decreased with increasing the ratio (runs 1–3). As shown in Figure 1a (shown below), rather linear relationships between the *M*_n_ values and the polymer yields (turnover numbers, TON) were observed, suggesting that these polymerizations proceeded in a (quasi) living manner.

It also turned out that the PDI values became low when the polymerization was conducted at −40 °C (run 4), with decreasing the activity [activity after 20 min: 4120 kg-polymer/mol-Ti·h (run 3, at −30 °C) vs. 1810 (run 4, at −40 °C)]. As shown in Figure 1a, a relatively good linear relationship between the *M*_n_ values and the polymer yields consistent with rather low PDI values (*M*_w_/*M*_n_ = 1.25–1.30) clearly suggest that the polymerization proceeded in a (quasi) living manner. Moreover, the PDI value became low (*M*_w_/*M*_n_ = 1.13–1.21) when the polymerization was conducted at −50 °C. The *M*_n_ value in the resultant polymers increased over time course consistent with low PDI values—high molecular weight poly(DC) with low PDI (*M*_n_ = 4.65 × 10^5^, *M*_w_/*M*_n_ = 1.15, run 5) was thus obtained after 2 h.

Similarly, as shown in Table 3, the polymerization of DD at −30 °C (runs 6–8) proceeded with high catalytic activities (5020–8950 kg-polymer/mol-Ti·h after 20 min) without significant catalyst deactivation to afford high molecular weight poly(DD)s (*M*_n_ = 4.84–6.74 × 10^5^), and the *M*_n_ values increased over time course without increasing the PDI (*M*_w_/*M*_n_) values. The PDI values decreased upon increasing the Al(*n*-C_8_H_17_)_3_/Al*^i^*Bu_3_ molar ratio, whereas the catalytic activity decreased with increasing the ratio. As also shown in Figure 1b (shown below), rather linear relationships between the *M*_n_ values and the polymer yields (turnover numbers, TON) suggest that these polymerizations also proceeded in a (quasi) living manner.

It was also revealed that the PDI values became low when the polymerization was conducted at −40 °C (run 9), although the catalytic activity decreased at −40 °C [activity after 20 min: 5020 kg-polymer/mol-Ti·h (run 8, at −30 °C) vs. 1720 (run 9, at −40 °C)]. As shown in Figure 1b, a relatively good linear relationship between *M*_n_ value and the polymer yield consistent with rather low PDI values (*M*_w_/*M*_n_ = 1.22–1.29) clearly suggests that the polymerization proceeded in a (quasi) living manner. Moreover, the PDI value became low (*M*_w_/*M*_n_ = 1.11–1.18) when the polymerization was conducted at −50 °C. The *M*_n_ value in the resultant polymers increased over time course consistent with low PDI values; high molecular weight poly(DD) with low PDI (*M*_n_ = 5.26 × 10^5^, *M*_w_/*M*_n_ = 1.14) could be thus obtained after 2 h.

As shown in Figure 1, good linear relationships between the *M*_n_ values and the polymer yields (TONs) were observed without increasing the PDI values in all cases, suggesting that these polymerizations proceeded in a (quasi) living manner. In particular, the polymerizations at −40 °C afforded polymers with low PDI values, the results thus strongly indicate a possibility of living polymerization under these conditions. The resultant poly(DD)s showed similar thermal property (melting temperature (*T*_m_) = −24 °C) to those prepared by Cp*TiCl_2_(O-2,6-*^i^*Pr_2_C_6_H_3_)–MAO catalyst [28], whereas *T*_m_ values increased upon increasing methylene units in the alkyl side chain (poly(1-hexadecane) = 26 °C, poly(1-octadecene = 42 °C) due to called side chain crystallization [28]. 

### 2.2. Polymerization of 1-Tetradecene by Cp*TiMe_2_(O-2,6-^i^Pr_2_C_6_H_3_) (1)–Borate Catalyst

**Figure 2:**
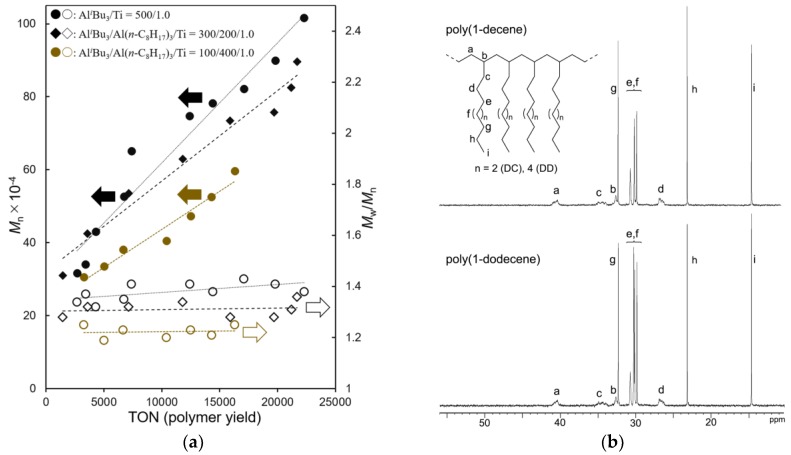
(**a**) Plots of *M*_n_, *M*_w_/*M*_n_ vs. polymer yields (turnover numbers, TON) in 1-tetradecene (TD) polymerization using Cp*TiMe_2_(O-2,6-*^i^*Pr_2_C_6_H_3_) (**1**)–borate catalyst. Detailed data are shown in Table 4. (**b**) ^13^C NMR spectrum (in CDCl_3_ at 25 °C) for poly(1-decene) (top, sample, run 1) and poly(1-dodecene) (bottom, sample, run 10).

Polymerizations of 1-tetradecene (TD) polymerizations using **1**–[Ph_3_C][B(C_6_F_5_)_4_] (borate) catalyst were conducted in *n*-hexane in the presence of Al cocatalyst with different Al*^i^*Bu_3_/Al(*n*-C_8_H_17_)_3_ molar ratios at −30 °C. In order to avoid freezing of the reaction mixture at −30 °C (due to melting temperature of TD, ca. −12 °C), rather diluted conditions compared to those for DC, DD polymerizations (TD 20 mL in *n*-hexane total 60 mL) were chosen. The results conducted under various Al*^i^*Bu_3_/Al(*n*-C_8_H_17_)_3_ molar ratios are summarized in Table 4. Plots of *M*_n_, *M*_w_/*M*_n_ vs. polymer yields (turnover numbers, TON) in the polymerization are also shown in Figure 2a.

It turned out that, as observed in polymerizations of DC and DD, these polymerizations proceeded without significant decreases in the catalytic activities (based on polymer yields), whereas the observed activity decreased with decreasing the Al*^i^*Bu_3_/Al(*n*-C_8_H_17_)_3_ molar ratios. The *M*_n_ values in the resultant polymers increased upon increasing the polymer yields (over time course) with consistent PDI values, and good linear relationships between the *M*_n_ values and the polymer yields were thus obtained, as shown in Figure 2a. These results clearly suggest that these polymerizations proceed in a (quasi) living manner. It also turned out that the *M*_n_ values after certain turnovers increased upon decreasing the Al*^i^*Bu_3_/Al(*n*-C_8_H_17_)_3_ molar ratios (eg. *M*_n_ = 7.83 × 10^5^ (14,400 turnovers, run 11) vs. *M*_n_ = 7.34 × 10^5^ (15,900 turnovers, run 12) vs. *M*_n_ = 5.26 × 10^5^ (14,300 turnovers, run 13)) along with decreasing the PDIs. The results thus suggest an increase of percentage of catalytically active species in situ upon increasing the ratio of Al(*n*-C_8_H_17_)_3_, although we could not estimate the exact number of catalytically active species at this moment (because the *M*_n_ values were estimated on gel-permeation chromatography (GPC) trace vs. polystyrene standards). Poly(TD) with ultrahigh molecular weight (*M*_n_ = 1.02 × 10^6^, *M*_w_/*M*_n_ = 1.38) could be thus obtained in the polymerization using **1**–borate-Al*^i^*Bu_3_ catalyst (run 11, after 2 h). 

Figure 2b shows selected ^13^C NMR spectra (in CDCl_3_ at 25 °C) for poly(DC) and poly(DD). It is clear that the resultant polymers do not have stereo-regularity (atactic polymers) [49], and as observed in the spectra in poly(1-hexene) [50], resonances ascribed to 2,1- or other insertion units could not be found. The results strongly suggest that these polymerizations proceeded with (in high certainty) 1,2-insertion manner.

## 3. Materials and Methods

All experiments were carried out under a nitrogen atmosphere in a Vacuum Atmospheres drybox unless otherwise specified. All chemicals used were of reagent grade and were purified by the standard purification procedures. Anhydrous grade of toluene (Kanto Kagaku Co. Ltd., Tokyo, Japan) was transferred into a bottle containing molecular sieves (mixture of 3A and 4A 1/16, and 13X) in the drybox, and was used without further purification. Reagent grades 1-decene (TCI Co., Ltd., Tokyo, Japan), 1-dodecene (TCI Co., Ltd.), 1-tetradecene (TCI Co., Ltd.) were stored in bottles in the drybox and were passed through an alumina short column prior to use. Syntheses of Cp*TiMe_2_(O-2,6-*^i^*Pr_2_C_6_H_3_) (**1**) was according to our previous report [50]. Ph_3_CB(C_6_F_5_)_4_ was purchased from Asahi Glass Co. Ltd., and was used as received in the drybox.

All ^1^H and ^13^C NMR spectra were recorded on a Bruker AV 500 spectrometer (500.13 MHz for ^1^H; 125.77 MHz for ^13^C), and all chemical shifts are given in ppm and are referred to SiMe_4_. ^13^C NMR spectra for the resultant polymers were recorded with proton decoupling, and the pulse interval was 5.2 s, the acquisition time was 0.8 s, the pulse angle was 90°, and the number of transients accumulated was about 6000. The polymer samples for analysis were prepared by dissolving the polymers in CDCl_3_ solution, and the spectra was measured at 25 °C. Molecular weights and the molecular weight distributions of the resultant polymers were measured by gel-permeation chromatography (GPC). HPLC grade THF was used for GPC and was degassed prior to use. GPC was performed at 40 °C on a Shimadzu SCL-10A using a RID-10A detector (Shimadzu Co. Ltd.) in THF (containing 0.03 wt. % of 2,6-di-*tert*-butyl-p-cresol, flow rate 1.0 mL/min). GPC columns (ShimPAC GPC-806, 804 and 802, 30 cm × 8.0 mm diameter, spherical porous gel made of styrene/divinylbenzene copolymer, ranging from <10^2^ to 2 × 10^7^ MW) were calibrated versus polystyrene standard samples. The molecular weight was calculated by a standard procedure based on the calibration with standard polystyrene samples.

Typical polymerization procedures were as follows: 1-decene (30 mL), *n*-hexane (30 mL) and a prescribed amount of Al*^i^*Bu_3_ [and Al(*n*-C_8_H_17_)_3_] was added into a 100 mL round-bottom flask connected to three-way valves under N_2_, the solution was then cooled to −30 °C. A toluene solution containing **1** (2.0 µmol/mL) [pre-treated with 2.0 eq. of Al*^i^*Bu_3_ at −30 °C] was added into the mixture, and the polymerization was then started by the addition of a prescribed amount of toluene solution containing Ph_3_CB(C_6_F_5_)_4_ (2.0 μmol/mL). A prescribed amount (3.0 mL) of the reaction mixture was removed via a syringe from the polymerization solution to monitor the time course, and the sample solution was then quickly poured into *^i^*PrOH (150 mL) containing HCl (10 mL). The resultant polymer was collected and was adequately washed with *^i^*PrOH and then dried *in* *vacuo*.

## 4. Conclusions

We have shown that polymerizations of 1-decene (DC), 1-dodecene (DD), and 1-tetradecene (TD) using Cp*TiMe_2_(O-2,6-*^i^*Pr_2_C_6_H_3_) (**1**)–[Ph_3_C][B(C_6_F_5_)_4_] (borate) catalyst proceeded with high catalytic activities, affording (ultra)high molecular weight polymers. The polymerizations of DC and DD, in *n*-hexane containing a mixture of Al*^i^*Bu_3_ and Al(*n*-C_8_H_17_)_3_ at −30 °C, proceeded with high catalytic activities (4120–5860 kg-poly(DC)/mol-Ti·h) without catalyst deactivation, affording high molecular weight polymers (*M*_n_ for poly(DC) = 7.04–7.82 × 10^5^ after 20 min). The PDI (*M*_w_/*M*_n_) values were affected by the ratio of Al(*n*-C_8_H_17_)_3_/Al*^i^*Bu_3_ as well as the polymerization temperature. Synthesis of high molecular weight poly(DD) with low PDI (*M*_n_ = 5.26 × 10^5^, *M*_w_/*M*_n_ = 1.16) could be attained at −50 °C. The TD polymerization using **1**–borate–Al*^i^*Bu_3_ catalyst (conducted in *n*-hexane at −30 °C) also afforded ultrahigh molecular weight poly(TD) (*M*_n_ = 1.02 × 10^6^, *M*_w_/*M*_n_ = 1.38). The results presented here are rare demonstrations for successful synthesis of (ultra)high molecular weight bottlebrush poly(α-olefin)s with narrow molecular weight distributions by polymerization of higher α-olefins, which proceeded in a (quasi) living manner. The fact should be important for synthesis of new polyolefins as well as design of efficient molecular catalysts for olefin polymerization.

## Figures and Tables

**Table 1:** Polymerization of 1-octene (OC) and 1-dodecene (DD), using Cp*TiX_2_(O-2,6-*^i^*Pr_2_C_6_H_3_) [X = Cl, Me (**1**)], [Me_2_Si(C_5_Me_4_)(N*^t^*Bu)]TiCl_2_, Cp_2_ZrCl_2_—cocatalyst systems [28]. *^a^*

Catalyst	α-Olefin	Cocatalyst	Time/min	TON *^b^*	Activity *^c^*	*M*_n_ *^d^*	*M*_w_ */M*_n_ *^d^*
Cp*TiCl_2_(O-2,6-*^i^*Pr_2_C_6_H_3_)	OC	MAO	30	9950	2240	501000	1.42
Cp*TiCl_2_(O-2,6-*^i^*Pr_2_C_6_H_3_)	DD	MAO	30	4120	1390	525000	1.35
Cp*TiMe_2_(O-2,6-*^i^*Pr_2_C_6_H_3_)	OC	borate	20	7080	2380	1970000	2.04
Cp*TiMe_2_(O-2,6-*^i^*Pr_2_C_6_H_3_)	DD	borate	20	3730	1880	1320000	1.99
[Me_2_Si(C_5_Me_4_)(N*^t^*Bu)]TiCl_2_	OC	MAO	30	4810	1080	292000	1.64
[Me_2_Si(C_5_Me_4_)(N*^t^*Bu)]TiCl_2_	DD	MAO	30	2060	690	351000	1.33
Cp_2_ZrCl_2_	OC	MAO	30	21300	4790	4100	1.55
Cp_2_ZrCl_2_	DD	MAO	30	15400	5190	5000	1.57

*^a^* Conditions (MAO cocatalyst): Complex 1.0 μmol, α-olefin 5.0 mL, d-MAO (prepared by removing toluene and AlMe_3_ from ordinary MAO) 2.0 mmol, 25 °C; Conditions (borate cocatalyst): Complex **1** 0.25 µmol, olefin 5.0 mL, −30 °C, Al*^i^*Bu_3_/[Ph_3_C][B(C_6_F_5_)_4_]/Ti = 500/3.0/1.0 (molar ratio). *^b^* TON (turnover number) = (mmol of α-olefin reacted)/(mmol-Ti). *^c^* Activity = kg-polymer/mol-Ti·h. *^d^* Gel-permeation chromatography (GPC) data THF vs. polystyrene standards.

**Table 2:** 1-Decene polymerization by Cp*TiMe_2_(O-2,6-*^i^*Pr_2_C_6_H_3_) (**1**)–borate catalyst. *^a^*

Run	Al(*n*-C_8_H_17_)_3_/Al*^i^*Bu_3_/Ti *^b^*	Temp./°C	Time/min	Polymer Yield *^c^*/mg	Activity *^d^*	TON *^e^*	*M*_n_*^f^* × 10^−^^4^	*M*_w_/*M*_n_*^f^*
1	-/500	–30	5	926	11110	6600	40.1	1.52
			10	1162	6970	8280	58.2	1.57
			15	1644	6580	11700	68.8	1.61
			20	1952	5860	13900	78.2	1.61
2	200/300	−30	5	366	4390	2610	39.6	1.45
			10	1130	6780	8050	61.3	1.43
			15	1454	5820	10360	68.7	1.50
			20	1708	5120	12170	76.4	1.52
3	400/100	−30	5	348	4180	2480	40.8	1.32
			10	752	4510	5360	58.0	1.36
			15	1240	4960	8840	64.2	1.38
			20	1372	4120	9780	70.4	1.37
4	400/100	−40	10	250	1500	1780	31.9	1.26
			15	408	1630	2910	39.0	1.30
			20	602	1810	4290	47.7	1.25
			30	976	1950	6960	56.9	1.25
			45	1228	1640	8750	63.4	1.29
			60	1620	1620	11500	66.8	1.29
5	300/200	−50	15	250	1000	1780	36.2	1.10
			30	490	980	3490	42.0	1.17
			45	506	680	3610	43.1	1.21
			60	534	530	3810	44.6	1.13
			75	558	450	4000	45.0	1.13
			90	582	390	4150	45.5	1.16
			105	652	370	4650	46.1	1.14
			120	684	340	4880	46.5	1.15

*^a^* Conditions: Complex **1** 1.0 µmol, 1-decene (30 mL) and *n*-hexane total 60 mL (initial 1-decene concentration 2.64 M), [Ph_3_C][B(C_6_F_5_)_4_]/Ti = 3.0 (molar ratio), **1** was pre-treated with 2.0 eq. of Al*^i^*Bu_3_ at −30 °C for 10 min. *^b^* Molar ratio of Al(*n*-C_8_H_17_)_3_/Al*^i^*Bu_3_/Ti. *^c^* A prescribed amount (3.0 mL) of the reaction mixture was removed via syringe from the mixture, and the yields were based on obtained amount. *^d^* Activity in kg-polymer/mol-Ti·h. *^e^* TON (turnovers) = molar amount of 1-decene consumed/mol·Ti. *^f^* GPC data in THF vs. polystyrene standards.

**Table 3:** 1-Dodecene polymerization by Cp*TiMe_2_(O-2,6-*^i^*Pr_2_C_6_H_3_) (**1**)–borate catalyst. *^a^*

Run	Al(*n*-C_8_H_17_)_3_/Al*^i^*Bu_3_/Ti *^b^*	Temp./°C	Time/min	Polymer Yield *^c^*/mg	Activity *^d^*	TON *^e^*	*M*_n_*^f^* × 10^−^^4^	*M*_w_/*M*_n_*^f^*
6	-/500	–30	5	600	7200	3570	34.8	1.58
			10	1236	7420	7340	48.0	1.66
			15	2288	9150	13600	59.2	1.60
			20	2982	8950	17700	67.4	1.68
7	200/300	–30	5	4480	5380	2660	32.9	1.52
			10	9300	5580	5530	41.2	1.53
			15	1752	7010	10400	48.8	1.58
			20	2094	6280	12400	55.4	1.61
8	400/100	–30	5	398	4780	2370	36.1	1.56
			10	668	4010	4000	45.4	1.52
			15	1204	4820	7150	44.7	1.52
			20	1674	5020	9950	48.4	1.54
9	400/100	–40	10	304	1820	1810	35.3	1.25
			15	428	1710	2540	39.3	1.22
			20	574	1720	3410	40.9	1.25
			30	654	1310	3890	42.7	1.26
			45	682	910	4050	43.1	1.27
			60	910	910	5410	45.2	1.29
10	250/250	–50	60	267	530	3810	25.5	1.18
			75	279	450	4000	31.2	1.17
			90	291	390	4150	40.8	1.11
			105	326	370	4650	48.1	1.18
			120	342	340	4880	52.6	1.14

*^a^* Conditions: Complex **1** 1.0 µmol, 1-dodecene (35 mL) and *n*-hexane total 60 mL (initial 1-dodecene concentration 2.63 M), [Ph_3_C][B(C_6_F_5_)_4_]/Ti = 3.0 (molar ratio), **1** was pre-treated with 2.0 eq. of Al*^i^*Bu_3_ at −30 °C for 10 min. *^b^* Molar ratio of Al(*n*-C_8_H_17_)_3_/Al*^i^*Bu_3_/Ti. *^c^* A prescribed amount (3.0 mL) of the reaction mixture was removed via syringe from the mixture, and the yields were based on obtained amount. *^d^* Activity in kg-polymer/mol-Ti·h. *^e^* TON (turnovers) = molar amount of 1-decene consumed/mol·Ti. *^f^* GPC data in THF vs. polystyrene standards.

**Table 4:** Polymerization of 1-tetradecene by Cp*TiMe_2_(O-2,6-*^i^*Pr_2_C_6_H_3_) (**1**)–borate catalyst (−30 °C). *^a^*

Run	Al(*n*-C_8_H_17_)_3_/Al*^i^*Bu_3_/Ti *^b^*	Time/min	Polymer Yield *^c^*/mg	Activity *^d^*	TON *^e^*	*M*_n_*^f^* × 10^−^^4^	*M*_w_/*M*_n_*^f^*
11	-/500	5	376	4510	2680	31.7	1.34
		10	480	2880	3420	34.1	1.37
		20	602	1810	4290	43.0	1.32
		30	944	1890	6730	52.7	1.35
		45	1034	1380	7370	65.1	1.41
		60	1734	1730	12400	74.8	1.41
		75	2016	1610	14400	78.3	1.38
		90	2396	1600	17100	82.2	1.43
		105	2778	1580	19800	90.0	1.41
		120	3132	1570	22300	101.6	1.38
12	200/300	20	202	610	1440	31.0	1.28
		30	502	1000	3580	42.5	1.32
		45	1000	1330	7130	53.5	1.32
		60	1662	1660	11800	62.9	1.34
		75	2232	1790	15900	73.4	1.28
		90	2738	1850	19700	75.7	1.28
		105	2980	1700	21200	82.5	1.31
		120	3044	1520	21700	89.6	1.36
13	400/100	30	458	920	3260	30.6	1.25
		45	702	940	5000	33.5	1.19
		60	934	930	6660	38.1	1.23
		75	1464	1170	10400	40.5	1.20
		90	1750	1170	12500	47.3	1.23
		105	2000	1140	14300	52.6	1.21
		120	2292	1150	16300	59.7	1.25

*^a^* Reaction conditions: Complex **1** 1.0 µmol, total volume of 1-tetradecene (20 mL) and *n*-hexane = 40 mL, [Ph_3_C][B(C_6_F_5_)_4_]/Ti = 3.0 (molar ratio), **1** was pre-treated with 2.0 eq. of Al*^i^*Bu_3_ at −30 °C for 10 min. *^b^* Molar ratio of Al(*n*-C_8_H_17_)_3_/Al*^i^*Bu_3_/Ti. *^c^* A prescribed amount (3.0 mL) of the reaction mixture was removed via syringe from the mixture, and the yields were based on obtained amount. *^d^* Activity in kg-polymer/mol-Ti·h. *^e^* TON (turnovers) = molar amount of 1-decene consumed/mol·Ti. *^f^* GPC data in THF vs. polystyrene standards.
